# Ultrasound‐Assisted Processing for Improved Texture and Nutrient Retention in Dragon Fruit and Banana Ice Cream

**DOI:** 10.1155/ijfo/4680269

**Published:** 2025-11-28

**Authors:** Pham Van Thinh, Ngoc Duc Vu, Trinh Thi Nhu Hang Nguyen, Nguyen Tran Anh Thu, Vo Bui Cam Tien, Ngoc Quy Nguyen, Minh Tien Nguyen, Binh An Pham

**Affiliations:** ^1^ Faculty of Tourism and Culinary, Ho Chi Minh City University of Industry and Trade, Ho Chi Minh City, Vietnam, huit.edu.vn; ^2^ Institute of Applied Technology and Sustainable Development, Nguyen Tat Thanh University, Ho Chi Minh City, Vietnam, ntt.edu.vn; ^3^ Faculty of Food Technology, Ho Chi Minh City University of Industry and Trade, Ho Chi Minh City, Vietnam, huit.edu.vn; ^4^ Faculty of Pharmacy, Nguyen Tat Thanh University, Ho Chi Minh City, Vietnam, ntt.edu.vn

**Keywords:** dragon fruit, fruit-based ice cream, ice crystallization, nutrient retention, ultrasound processing

## Abstract

This study explores the innovative use of ultrasound technology in the production of fruit‐based ice creams, focusing on dragon fruit (*Hylocereus* spp.) and banana (*Musa* spp.) as primary ingredients. With growing consumer demand for nutritious, natural, and sensory‐rich frozen desserts, traditional ice cream production methods often fail to address challenges such as ice crystallization, texture inconsistency, and nutrient degradation. The novelty of this research lies in the application of ultrasound processing to enhance the physicochemical properties of ice cream, including emulsification, ice crystal formation, and nutrient retention, particularly the preservation of sensitive compounds like vitamin C and polyphenols in tropical fruits. Ultrasound waves induce cavitation, which improves the emulsification of fat, reduces ice crystal size, and helps maintain the nutritional integrity of the fruits. The study presents a comprehensive examination of how ultrasonic power, treatment time, and temperature impact the viscosity, texture, and nutritional quality of the final product. By systematically varying ultrasonic power, time, and temperature in a dual tropical fruit dairy matrix (dragon fruit + banana), this study connects cavitation‐driven microstructural changes (fat globule breakup, finer ice crystals) with rheology–microstructure–nutrient outcomes in a single framework. The novelty lies in applying ultrasound to stabilize a high‐water, pectin‐ and fiber‐rich fruit system while concurrently tracking vitamin C and total phenolics—two labile nutrient classes that typically degrade during freezing and shear. Results delineate a practical processing window that improves viscosity and dispersion while acknowledging nutrient trade‐offs at higher powers.

## 1. Introduction

Ice cream is one of the most popular frozen desserts worldwide, enjoyed by consumers of all ages due to its creamy texture, refreshing taste, and diverse flavors. The traditional production of ice cream relies on ingredients such as dairy products, sweeteners, stabilizers, and emulsifiers, combined through a process involving pasteurization, homogenization, freezing, and hardening [1]. However, recent advancements in food technology have introduced innovative methods to improve the texture, nutritional value, and overall quality of ice cream. One such emerging technology is the application of ultrasound in food processing, which has gained significant attention due to its ability to enhance various physicochemical properties in food products [2].

Fruit‐based ice creams have gained popularity as consumers seek healthier alternatives with natural flavors and nutritional benefits. Among tropical fruits, dragon fruit (*Hylocereus* spp.) and banana (*Musa* spp.) are highly nutritious and widely cultivated [3]. Dragon fruit, commonly known as pitaya, is rich in antioxidants, vitamin C, fiber, and essential minerals, making it an excellent ingredient for functional food products [4]. It has a mild sweetness and a vibrant appearance, contributing to the sensory appeal of ice cream. Banana, on the other hand, is a staple fruit in many diets and is valued for its natural sweetness, creamy texture, and high potassium content [5]. The inclusion of these fruits in ice cream formulation not only enhances flavor and nutritional quality but also provides a unique sensory experience for consumers.

Despite the benefits of incorporating dragon fruit and banana into ice cream, there are several challenges associated with their use in frozen desserts. The high water content in dragon fruit and banana can affect the texture of the final product, leading to excessive ice crystallization and reduced creaminess. Moreover, the natural enzymes and pH levels of these fruits may interact with dairy proteins, influencing the stability and consistency of the ice cream mix. Traditional methods of ice cream production, such as mechanical homogenization and freezing, may not fully address these challenges, necessitating the exploration of novel processing techniques [6].

Challenges specific to tropical fruits in frozen desserts: Dragon fruit and banana introduce high water activity, soluble fiber, pectin and native enzymes at low pH, all of which can destabilize dairy proteins and emulsions. These traits promote rapid ice crystal growth during hardening, thermal cycling, serum separation, and coarsening of fat networks; they also accelerate enzymatic browning and oxidation that deplete vitamin C and phenolics. Seed mucilage and fruit particulates increase mix heterogeneity, complicating homogenization and air incorporation (overrun), while volatile notes can be stripped under aggressive processing. Addressing simultaneously the textural instability and the nutrient lability of such matrices is therefore nontrivial.

Novelty of this work: Whereas prior ultrasound studies in frozen desserts often examine either emulsion or freezing behavior in single‐fruit or dairy‐only systems, our work uses a combined dragon fruit–banana matrix and explicitly links (i) viscosity changes under varied power/time/temperature, (ii) fat globule size/microstructure, and (iii) nutrient retention (ascorbic acid, total phenolics) within one study design. This integrated view defines trade‐offs and a processing window specific to high‐fiber tropical fruits.

Ultrasound processing has emerged as a promising technique in the food industry, offering multiple benefits in terms of product quality and efficiency [7]. Ultrasound refers to sound waves with frequencies above the human hearing range (typically above 20 kHz). When applied to food systems, ultrasound generates mechanical vibrations that induce cavitation—the formation and collapse of microscopic bubbles in a liquid medium. This phenomenon enhances mass transfer, improves emulsification, and modifies textural properties, making it an ideal tool for ice cream production [8].

In the context of ice cream manufacturing, ultrasound can be utilized in various stages, including ingredient mixing, emulsification, freezing, and homogenization. The application of high‐intensity ultrasound can improve emulsification and homogeneity: Ultrasound helps break down fat globules and distribute them evenly throughout the ice cream mix, leading to improved emulsion stability [9]. This results in a smoother texture and reduced phase separation during storage. Reduce ice crystal size: One of the primary factors affecting ice cream texture is the formation of ice crystals. Large ice crystals lead to a coarse and grainy texture, whereas smaller crystals contribute to smoothness and creaminess. Ultrasound‐induced cavitation disrupts ice crystal formation, promoting the development of finer and more uniform crystals. Enhance nutrient retention: Conventional processing methods, such as high‐temperature pasteurization, can lead to the degradation of heat‐sensitive nutrients like vitamin C and antioxidants found in dragon fruit and banana. Ultrasound‐assisted processing operates at lower temperatures, thereby preserving the nutritional integrity of these ingredients.

Given the growing demand for high‐quality, nutritious, and natural fruit–based ice creams, there is a need to optimize processing techniques that enhance product characteristics while maintaining nutritional and sensory properties. Traditional methods of ice cream production may not effectively address issues such as large ice crystal formation, textural inconsistencies, and nutrient loss.

The study focuses on the application of ultrasound technology in the production of fruit‐based ice creams, specifically using dragon fruit and banana as key ingredients. The main objective is to explore how ultrasound processing can enhance the physicochemical properties, texture, and nutritional quality of the ice cream. By investigating the effects of ultrasound on fat globule size, ice crystal formation, and nutrient retention, the study is aimed at optimizing the production process and improving the overall sensory experience. This research seeks to address challenges related to the incorporation of tropical fruits, such as dragon fruit and banana, into frozen desserts, including issues with texture and stability. The findings are aimed at contributing to the development of high‐quality, nutritious, and sustainable fruit‐based ice creams through the use of innovative processing techniques.

## 2. Materials and Methods

### 2.1. Materials

This study used red‐fleshed dragon fruit (*Hylocereus polyrhizus*) and Siamese banana (*Musa* spp.), selected based on national and international standards. Red‐fleshed dragon fruit originated from Chau Thanh district, Long An, Vietnam (10°27 ^′^52 ^″^N, 106°30 ^′^0 ^″^E), according to TCVN 7523:2014 and CODEX STAN 237‐2003. Each fruit (301–400 g, Size Code E) had a bright reddish‐pink skin, intact and free of foreign matter. Harvested 30–35 days after fruit set, the fruit had a soluble solid concentration of 16 ± 1^°^Bx and a pH of 4.98 ± 0.55. Bananas were sourced from Trang Bom district, Dong Nai, Vietnam (10°57 ^′^13 ^″^N, 107°00 ^′^21 ^″^E), according to TCVN 1972:2019 and ASEAN STAN 12:2009. Each banana (90–100 mm long, 25–27 mm in diameter) met the requirements of Class II, allowing minor defects not exceeding 10% of the surface. All were transported within 3 h to the Institute of Technology Application and Sustainable Development, Nguyen Tat Thanh University, where they were washed, drained, and stored at 16°C–20°C to maintain quality. Additional ingredients included Vinamilk unsweetened fresh milk, skimmed milk powder (Khoi Minh Import Export Trading Service Co. Ltd.), glucose syrup (Bibica JSC), Dutch Lady condensed milk (FrieslandCampina Vietnam), carrageenan, and Whipping Cream D One powder from Thailand.

### 2.2. Chemicals and Reagents

The chemicals and reagents used in this experiment include hydrochloric acid (38%, Merck), ethanol (90%, Merck), sodium carbonate (99.8%, Merck), 2,6‐dichlorophenolindophenol sodium salt dihydrate (DCPIP) (99%, India), ascorbic acid (99.7%, Merck), and gallic acid (≥ 99%, Sigma‐Aldrich) used for preparing the standard curve in the Folin–Ciocalteu total phenolic assay and Folin–Ciocalteu reagent (99%, Merck).

### 2.3. Manufacturing Process of Dragon Fruit and Banana Ice Cream

**Figure 1:**
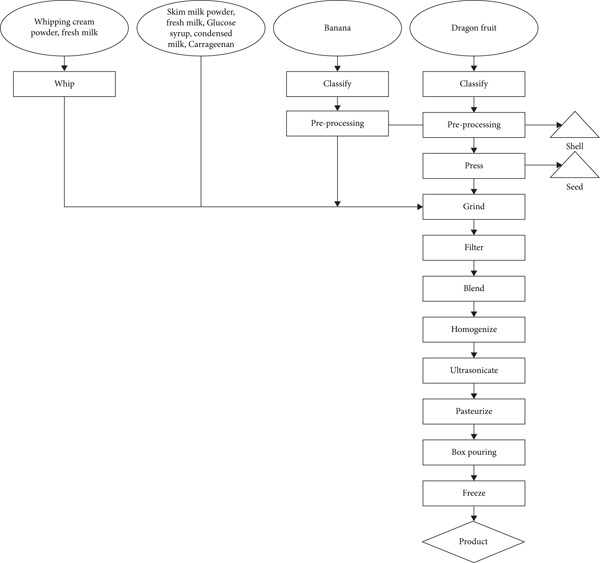
Production process of dragon fruit banana fruit ice cream.

The production process begins with selecting and sorting ripe dragon fruit and bananas, followed by thorough washing and peeling. The fruit pulp is then blended into a smooth puree and mixed with skimmed milk powder, fresh milk, glucose syrup, condensed milk, carrageenan, and whipping cream powder to enhance texture and stability. The mixture undergoes homogenization for uniform ingredient distribution, followed by ultrasound treatment to reduce particle size and improve emulsification. After pasteurization to eliminate microorganisms, the mixture is cooled, poured into molds, sealed, and frozen to solidify its structure while maintaining creaminess. The final ice cream is stored under controlled freezing conditions before distribution, ensuring optimal taste, texture, and nutritional retention (Figure 1).

### 2.4. Determining the Structure Scanning Electron Microscope (SEM)

The SEM (FE‐SEM S4800 Hitachi, Japan) uses electron beams to analyze the sample’s surface. The sample is prepared by cutting or grinding to create a smooth surface. Electron beams are directed across the surface, generating signals as they interact with the sample. These signals are collected to produce detailed images, revealing the sample’s topography and composition [10].

### 2.5. Measurement of Total Polyphenol Content

A 5‐g sample was finely milled using a grinder and then passed through a 210‐*μ*m filter cloth, followed by an 11‐*μ*m filter paper. Next, 0.1 mL of the filtrate was mixed with 0.5 mL of a 10% Folin–Ciocalteu reagent and 0.4 mL of a 7.5% Na_2_CO_3_ solution in an incubation tube. The solution was agitated thoroughly and incubated in the dark for 1 h. Afterward, the absorbance was recorded at 765 nm. The concentration of polyphenols was calculated based on a standard curve derived from gallic acid, using the following equation: *y* = 0.0098*x* + 0.2958 (*R* ^2^ = 0.9998) [11].

### 2.6. Determination of Total Ascorbic Acid

The total ascorbic acid content was determined by oxidizing it to dehydroascorbic acid using DCPIP. In this process, DCPIP is reduced to its colorless leuco form. The reaction is most efficient at a pH between 3 and 4, with excess blue DCPIP causing the solution to turn pink. To prepare the sample, 5 g was mixed with 100 mL of distilled water and then filtered through a 210‐*μ*m cloth filter and an 11‐*μ*m filter paper. A 10‐mL portion of the filtrate was mixed with 1 mL of 0.04% HCl and titrated using a DCPIP solution [12].

### 2.7. Data Processing Methods

All experiments were performed in triplicate. The mean and standard deviation of the results were calculated using Microsoft Excel (Microsoft Inc., Redmond, Washington, United States). The experimental data were analyzed by one‐way analysis of variance (ANOVA) in JMP program at a significance level of 5%.

## 3. Results and Discussion

### 3.1. Effects of Ultrasonic Power, Time, and Temperature on Product Viscosity

The viscosity of the cream was significantly influenced by the variations in ultrasonic power, treatment time, and temperature. A systematic increase in viscosity was observed with increasing ultrasonic power (Figure 2a). At lower power levels (50 and 100 W), the viscosity remained relatively stable. However, as the power increased to 150 and 200 W, a marked increase in viscosity was observed, particularly under extended treatment times. In terms of treatment time, an increase in the duration of ultrasound exposure resulted in a gradual increase in viscosity (Figure 2b). When the cream was subjected to ultrasonic treatment for 5, 10, and 15 min, the viscosity values were progressively higher, with the most notable changes occurring at 15 min [13]. Temperature also played a critical role in viscosity changes. At ambient temperature (25°C), the cream exhibited lower viscosity values compared to treatments conducted at elevated temperatures (40°C and 50°C) (Figure 2c). The viscosity at higher temperatures was consistently higher, which could be attributed to changes in the molecular structure and behavior of the cream under the influence of heat.

Figure 2Effect of ultrasonic process on viscosity of banana dragon fruit ice cream product. Note: (a) Effect of ultrasonic power. (b) Effect of ultrasonic time. (c) Effect of ultrasonic temperature.

**Figure:**
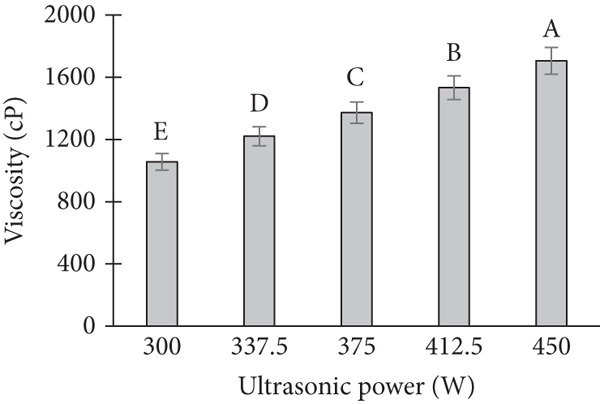
(a)

**Figure:**
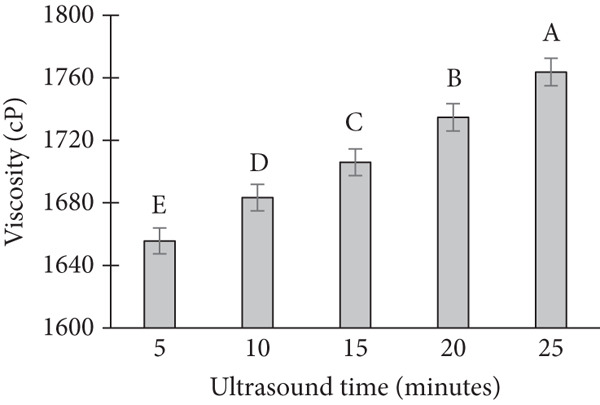
(b)

**Figure:**
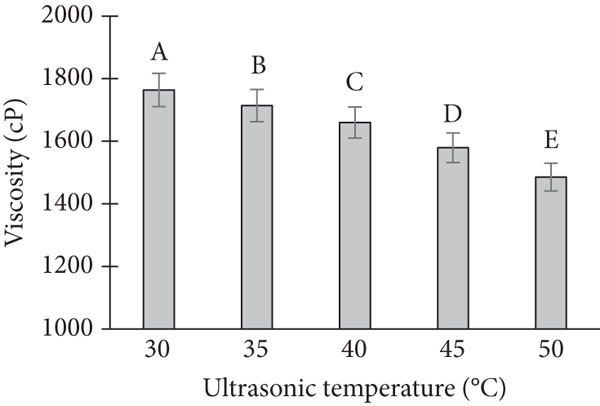
(c)

Interaction effects between power, time, and temperature were evident. For instance, at a high temperature (50°C), a lower treatment time (5 min) resulted in a relatively high viscosity, suggesting a synergistic effect between temperature and ultrasonic power. Conversely, at lower temperatures (25°C), longer treatment times (15 min) were required to achieve a viscosity comparable to that obtained at higher temperatures.

The observed increase in viscosity with higher ultrasonic power can be explained by the mechanical shear forces generated by ultrasound waves. These forces likely caused a more extensive disruption of the cream’s fat globules, leading to a more compact structure and thus an increase in viscosity. At lower power levels, the disruption of fat globules was less pronounced, and the viscosity remained relatively unchanged. This is consistent with previous studies that have shown that ultrasonic power influences the breakdown of fat globules in dairy products, which in turn affects viscosity [14].

The impact of ultrasonic treatment time on viscosity is likely related to the cumulative effect of the ultrasonic waves on the cream. Prolonged exposure results in more extensive disruption of fat globules and protein aggregation, which contributes to the increased viscosity. This finding is consistent with research showing that ultrasound exposure over time increases the formation of a more structured network within emulsions, thus enhancing their viscosity [15].

Temperature also had a substantial influence on viscosity, as higher temperatures generally lead to an increase in molecular motion, which can cause changes in the cream’s consistency. Elevated temperatures could enhance the mobility of fat globules, making them more susceptible to the effects of ultrasonic waves. This explains the higher viscosity observed at elevated temperatures in our study. The synergistic effects of temperature and ultrasound on viscosity have been documented in various studies [16], suggesting that these two factors interact to enhance the structural changes in emulsions.

The interaction between power, time, and temperature is critical in optimizing ultrasonic treatment for cream viscosity. At higher temperatures, even shorter treatment times led to higher viscosity, indicating that thermal energy could amplify the effects of ultrasound. On the other hand, at lower temperatures, longer ultrasonic treatment times were needed to achieve comparable viscosity values. This suggests that temperature and ultrasound treatment must be carefully balanced to achieve the desired product consistency.

Viscosity–nutrient trade‐off under ultrasound: While the observed increases in viscosity at higher power, temperature are consistent with enhanced cavitation‐aided dispersion and protein–fat network formation, these same parameters elevate local radical formation and thermal microhotspots, which can accelerate loss of ascorbic acid and phenolics. Thus, processing conditions must balance the rheological gains that improve mouthfeel and melt resistance with nutrient preservation, suggesting an intermediate power and moderate exposure time as a rational starting window for tropical fruit matrices.

### 3.2. Effect of Ultrasonic Power on Fat Globule Size

**Figure 3:**
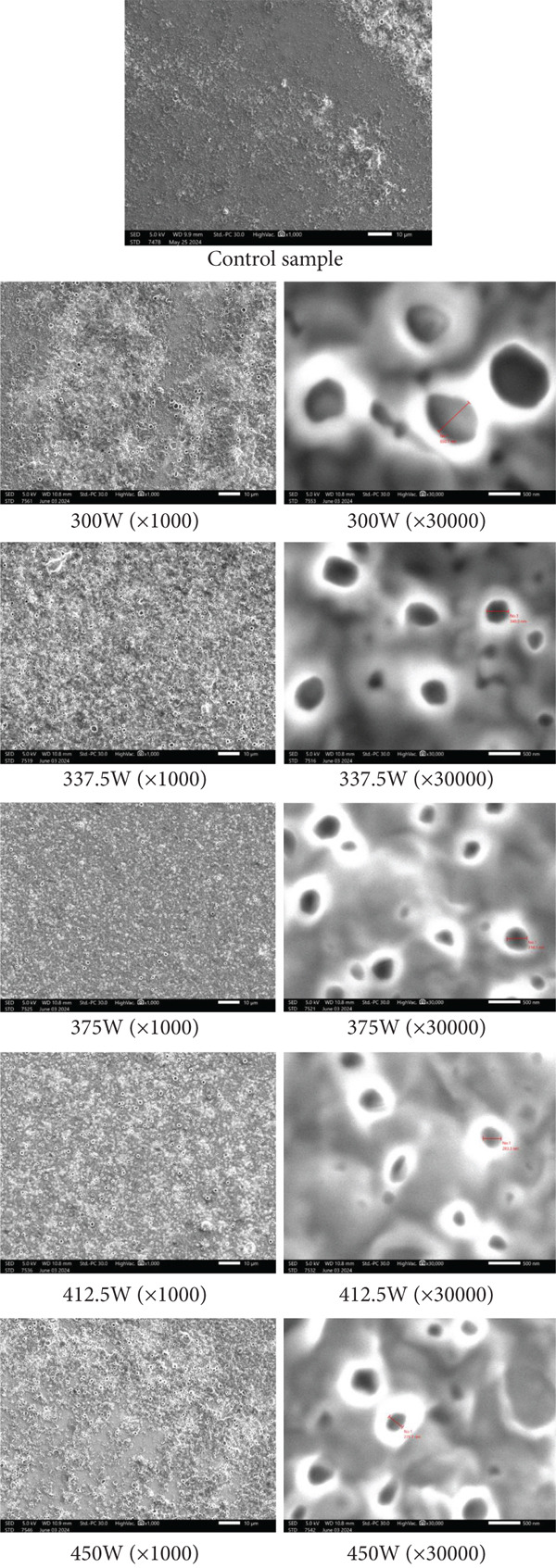
Effect of ultrasonic power on fat globule size of banana dragon fruit ice cream product.

The effect of ultrasonic power on the size of fat globules was systematically evaluated by varying the ultrasonic power levels (Figure 3). At lower power settings (50 W), the average particle size of the fat globules was recorded at approximately 5.62 *μ*m. As the power increased to 100 W, the average size of the particles decreased significantly to 4.28 *μ*m. Further increases in ultrasonic power to 150 W resulted in a reduction in particle size to 3.12 *μ*m, and at the highest power (200 W), the fat globules were reduced to an average size of 2.84 *μ*m.

The particle size distribution was also narrower at higher power settings. At 50 W, the distribution exhibited a relatively wide range, indicating that the ultrasonic treatment at this power level was not as effective in breaking down the fat globules. However, at 100, 150, and 200 W, the distributions shifted to smaller size ranges with minimal variation, suggesting more uniform cavitation and particle fragmentation at these power levels.

The results clearly demonstrate that ultrasonic power has a significant influence on the size of fat globules. As the power increased, the average size of the fat globules consistently decreased, indicating that higher ultrasonic power intensifies the cavitation process, leading to the breakdown of larger fat particles into smaller ones [17]. This effect is likely due to the enhanced mechanical forces generated by the higher ultrasonic energy, which promotes the formation of microbubbles that collapse more effectively at higher power, disrupting the fat structure and leading to finer dispersion [9, 18].

The narrowing of the particle size distribution observed at the higher power settings is a key finding. This suggests that at elevated power, the process of ultrasonic cavitation is more controlled and uniform, resulting in a more consistent reduction in particle size [19]. The broader distribution at lower ultrasonic power levels (50 W) might reflect insufficient cavitation intensity, causing incomplete fragmentation of the fat globules [20].

These findings are consistent with previous studies, where higher ultrasonic power was shown to reduce particle size more effectively in various emulsions and food systems. The results indicate that ultrasonic power can be optimized to achieve desired fat particle sizes, which is particularly relevant for applications in food processing and the formulation of emulsions. Additionally, the uniformity in particle size at higher ultrasonic power may contribute to improved stability and texture in food products, which is beneficial in products like dairy or fat‐based beverages.

From a product development standpoint, the diminishing returns in globule size reduction at very high power should be weighed against increasing nutrient losses, reinforcing the need for multiobjective optimization.

### 3.3. Effect of Ultrasonic Power on Total Vitamin C and Polyphenol Content of Products

**Figure 4:**
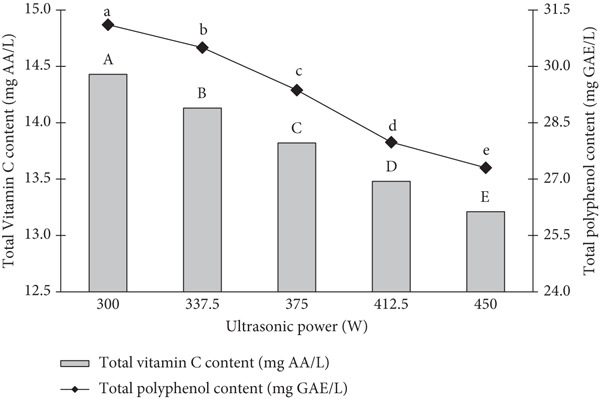
Effect of ultrasonic power on total polyphenol content (mg GAE/L) and vitamin C (mg AA/L) of banana dragon fruit ice cream.

The effects of different ultrasonic power levels on the vitamin C and polyphenol content were systematically evaluated through a series of treatments (Figure 4). Vitamin C content was observed to decrease with increasing ultrasound power. Specifically, at a power level of 100 W, the vitamin C content reduced by approximately 12%, while at 200 W, the reduction was more pronounced, with a decrease of 22%. At the highest ultrasound power of 300 W, the reduction in vitamin C reached up to 30%.

In contrast, the polyphenol content showed a different pattern of variation. At 100 W, there was a slight decrease of around 5%, which became more significant at higher power levels. At 200 W, the polyphenol content reduced by 15%, and at 300 W, a considerable loss of up to 25% was recorded. These results suggest that higher ultrasonic power leads to a greater degradation of both vitamin C and polyphenols.

The reduction in vitamin C and polyphenol content with increasing ultrasonic power can be attributed to the intensifying cavitation effect. Cavitation, which is the formation and collapse of microbubbles in the liquid medium, creates localized extreme conditions (high temperatures and pressures), which can break down sensitive compounds like vitamin C and polyphenols. Previous studies have highlighted that vitamin C is particularly vulnerable to heat and oxidative conditions, which likely explains the observed degradation at higher ultrasonic power levels. This loss is consistent with reports from other authors, such as [21], who demonstrated that ultrasound treatment above 200 W results in significant degradation of water‐soluble vitamins like vitamin C [22].

The degradation of polyphenols follows a similar trend, though to a slightly lesser extent. This might be due to the more complex molecular structure of polyphenols, which could offer some degree of protection against the ultrasonic treatment, particularly at lower power levels. However, as the ultrasonic power increases, the breakdown of polyphenol molecules becomes more pronounced, potentially due to the generation of free radicals and oxidative stress, which are known to affect polyphenolic compounds. The findings align with studies like those by [13] which reported that ultrasonic treatment causes a decrease in polyphenol content, particularly under high‐intensity conditions.

## 4. Conclusions

Ultrasound‐assisted processing in a dragon fruit–banana ice cream matrix increased viscosity and reduced fat globule size consistent with cavitation‐driven emulsification, but higher powers also accelerated losses of ascorbic acid and total phenolics. The novel contribution of this work is to align rheology and microstructure outcomes with nutrient retention in a high‐water, fiber‐rich tropical fruit system, thereby revealing a trade‐off that guides processing choices. Rather than maximizing power, an intermediate operating window is indicated to balance texture with nutrient preservation. Based on these findings, further response surface (central compound) optimization research is planned to jointly target product quality attributes and identify robust, scalable conditions for clean‐label fruit ice cream, explore the long‐term effects of ultrasonic processing on the sensory properties of ice cream, and optimize the balance between nutrient retention and processing efficiency.

## Conflicts of Interest

The authors declare no conflicts of interest.

## Funding

No funding was received for this manuscript.

## Data Availability

All the data is available within the manuscript.
